# Extracellular volume quantification by cardiac magnetic resonance imaging without hematocrit sampling: Ready for prime time?

**DOI:** 10.1007/s00508-017-1267-y

**Published:** 2017-10-04

**Authors:** Andreas A. Kammerlander, Franz Duca, Christina Binder, Stefan Aschauer, Caroline Zotter-Tufaro, Matthias Koschutnik, Beatrice A. Marzluf, Diana Bonderman, Julia Mascherbauer

**Affiliations:** 1 Department of Cardiology, Medical University of Vienna, Waehringer Guertel 18–20, 1090 Vienna, Austria; 2 Otto Wagner Hospital, Wien, Austria

**Keywords:** Cardiovascular magnetic resonance imaging, Extracellular volume, T1-mapping, Modified look-locker inversion recovery, Synthetic hematocrit

## Abstract

**Background:**

Myocardial tissue characterization by cardiovascular magnetic resonance (CMR) T1 mapping currently receives increasing interest as a diagnostic tool in various disease settings. The T1-mapping technique allows non-invasive estimation of myocardial extracellular volume (ECV) using T1-times before and after gadolinium administration; however, for calculation of the myocardial ECV the hematocrit is needed, which limits its utility in routine application. Recently, the alternative use of the blood pool T1-time instead of the hematocrit has been described.

**Methods:**

The results of CMR T1 mapping data of 513 consecutive patients were analyzed for this study. Blood for hematocrit measurement was drawn when placing the i. v. line for contrast agent administration. Data from the first 200 consecutive patients (derivation cohort) were used to establish a regression formula allowing synthetic hematocrit calculation, which was then validated in the following 313 patients (validation cohort). Synthetic ECV was calculated using synthetic hematocrit, and was compared with conventionally derived ECV.

**Results:**

Among the entire cohort of 513 patients (mean age 57.4 ± 17.5 years old, 49.1% female) conventionally measured hematocrit was 39.9 ± 4.7% and native blood pool T1-time was 1570.6 ± 117.8 ms. Hematocrit and relaxivity of blood (R1 = 1/blood pool T1 time) were significantly correlated (r = 0.533, r^2^ = 0.284, *p* < 0.001). By linear regression analysis, the following formula was developed from the derivation cohort: synthetic hematocrit = 628.5 × R1 − 0.002. Synthetic and conventional hematocrit as well as ECV showed significant correlation in the validation (r = 0.533, r^2^ = 0.284, *p* < 0.001 and r = 0.943, r^2^ = 0.889, *p* < 0.001, respectively) as well as the overall cohort (r = 0.552, r^2^ = 0.305, *p* < 0.001 and r = 0.957, r^2^ = 0,916, *p* < 0.001). By Bland Altman analysis, good agreement between conventional and synthetic ECV was found in the validation cohort (mean difference: 0.007%, limits of agreement: −4.32 and 4.33%, respectively).

**Conclusion:**

Synthetic ECV using native blood pool T1-times to calculate the hematocrit, is feasible and leads to almost identical results in comparison with the conventional method. It may allow fully automatic ECV-mapping and thus enable broader use of ECV by CMR T1 mapping in clinical practice.

## Introduction

Over the last decade cardiovascular magnetic resonance imaging (CMR) has gained increasing importance in clinical practice. While echocardiography remains the standard cardiac imaging tool due to its broad availability and low cost, CMR is now the recognized reference method for evaluation of left and right ventricular size and function [1]. In addition, CMR allows visualization of focal fibrosis and focal expansion of the extracellular space through late gadolinium-enhanced (LGE) imaging [2]; however, LGE does not allow a quantitative analysis of diffuse fibrosis, which may affect large parts of the myocardium. By using recently described T1-mapping methods it is now possible to non-invasively calculate the extracellular volume (ECV), [3, 4], which has been shown to accurately reflect diffuse myocardial fibrosis when compared with biopsy samples [4–14]. The use of T1-mapping and ECV have been investigated in a broad range of cardiovascular diseases, including valvular heart disease [4, 7, 8, 13], cardiac amyloidosis [15, 16], myocarditis [17, 18], myocardial infarction [19], Anderson-Fabry disease [20] and heart failure [21, 22]. Several studies reported a strong association of ECV with stages of disease and cardiovascular outcomes [11, 23–25]; however, the hematocrit is needed to calculate the ECV, which limits its routine application and automatic post-scanning processing.

Recently, Treibel et al. demonstrated a linear relationship of hematocrit and blood relaxivity (R1 = 1/blood pool T1 time), which was used to develop a synthetic hematocrit and ECV [25]. The authors postulated that synthetic ECV allows reliable noninvasive quantification of myocardial ECV without blood sampling. So far, that is the only working group that has evaluated the accurateness of synthetic ECV. The aim of the present study was to develop and test a formula for the calculation of synthetic ECV at our center.

## Patients, material and methods

A total of 513 patients from our prospective T1-mapping registry were included in this study. All patients provided written informed consent. The ethics committee approved both the registry as well as the current study protocol.

### Cardiovascular magnetic resonance imaging

**Fig. 1:**
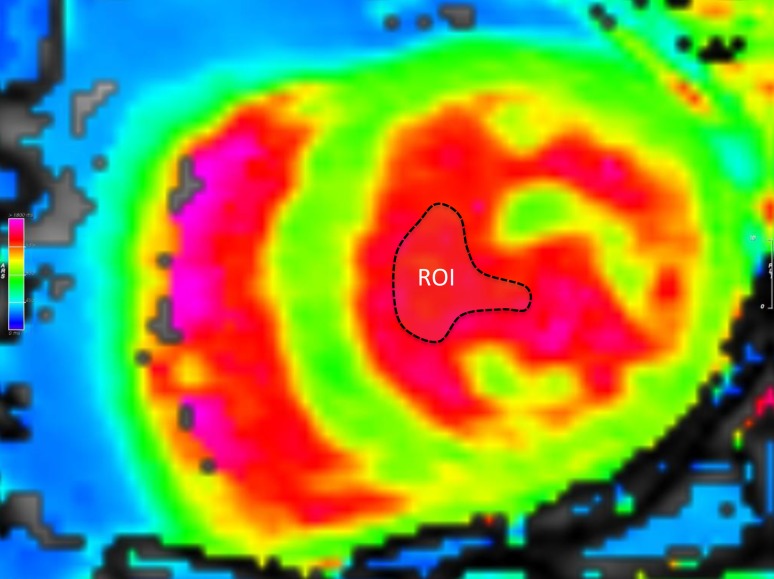
Measurement of T1 values of left ventricular blood pool. A mid-ventricular T1-map was chosen as region of interest (ROI *dashed line*) with adequate distance to endomyocardial borders and papillary muscles

At the day of CMR, demographic data as well as comorbidities, as listed in Table 1, were assessed. The CMR protocols consisted of standard functional studies including LGE. All CMR studies were performed on the same 1.5-T scanner (Magnetom Avanto, Siemens Healthcare, Erlangen, Germany). For T1-mapping, a modified look-locker inversion recovery (MOLLI) 5(3)3 sequence was used. The T1-maps were acquired both pre-administration and post-administration of 0.1 mmol/kg gadobutrol (Gadovist, Bayer Vital, Leverkusen, Germany) in a short axis and four-chamber view. The T1-sequence parameters were: starting inversion time (TI) 120 ms, TI increment 80 ms, reconstructed matrix size 256 × 218, measured matrix size 256 × 144 (phase-encoding resolution 66%, phase-encoding field of view 85%). Left ventricular (LV) myocardium and blood pool of the LV were measured in both views and results were averaged. Fig. 1 displays a native T1-map with the regions of interest (ROI).

**Table 1:** Baseline characteristics stratified by derivation and validation cohort

	All patients *n* = 513	Derivation cohort *n* = 200 (39.0%)	Validation cohort *n* = 313 (61.0%)	*p*-value
Clinical parameters				
Age (years)	57.4 ± 17.5	57.7 ± 17.3	57.3 ± 17.7	0.986
Female (%)	49.1	50.0	48.6	0.751
BMI (kg/m^2^)	27.3 ± 5.6	27.4 ± 5.8	27.2 ± 5.4	0.845
Hypertension (%)	68.9	68.3	69.3	0.821
Atrial fibrillation (%)	28.2	24.1	31.0	0.120
Diabetes (%)	16.4	16.3	16.5	0.959
CAD (%)	25.8	32.6	21.0	**0.007**
Previous PCI (%)	10.6	12.1	9.5	0.401
Previous CABG (%)	4.0	5.1	3.2	0.318
Previous MI (%)	9.6	13.6	6.7	**0.017**
Previous stroke (%)	3.7	4.0	3.6	0.819
Conventional hematocrit (%)	39.9 ± 4.7	40.2 ± 4.4	39.9 ± 4.8	0.490
eGFR (ml/min/1.73 m^2^)	78.0 ± 25.9	80.1 ± 27.8	76.5 ± 24.5	0.187
Serum NT-proBNP (pg/ml)	1493.9 ± 3884.2	1319.4 ± 3583.5	1611.3 ± 4076.8	0.126
*Referral diagnosis*				0.076
Heart failure (%)	48.0	48.5	47.6	–
VHD (%)	18.3	14.5	20.8	–
CAD (%)	10.7	14.5	8.3	–
Others (%)	23.0	22.5	23.3	–
Cardiac magnetic resonance imaging parameters				
LA diameter	59.3 ± 10.1	58.5 ± 9.8	59.9 ± 10.3	0.099
RA diameter	59.4 ± 9.6	58.4 ± 8.6	60.0 ± 10.2	0.082
IVS (mm)	11.7 ± 3.3	11.8 ± 2.9	11.7 ± 3.5	0.426
LV mass (g)	119.8 ± 46.7	118.2 ± 38.4	121.1 ± 52.2	0.619
LVEF (%)	61.7 ± 11.8	62.2 ± 10.5	61.4 ± 12.5	0.730
LVEDVi (ml)	76.9 ± 24.6	74.5 ± 23.6	78.4 ± 25.2	0.066
Cardiac index (l/min)	3.1 ± 0.9	3.1 ± 0.9	3.1 ± 0.9	0.438
RVEF (%)	55.9 ± 10.1	56.4 ± 9.2	55.7 ± 10.7	0.499
RVEDVi (ml)	76.9 ± 20.7	76.6 ± 20.4	77.1 ± 21.0	0.834
LGE (% of LV mass)^a^	4.0 ± 7.3	4.3 ± 7.4	3.8 ± 7.2	0.729
Native myocardial T1-time (ms)	990.8 ± 55.3	991.4 ± 54.5	990.4 ± 56.0	0.151
Native blood T1-time (ms)	1570.6 ± 117.8	1561.4 ± 101.8	1576.5 ± 126.7	0.133
Conventional ECV (%)	28.4 ± 6.8	28.8 ± 7.8	28.2 ± 6.1	0.227

Values are mean ± SD or %

*BMI* indicates body mass index, *CAD* coronary artery disease, *PCI* percutaneous coronary intervention, *CABG* coronary artery bypass graft surgery, *MI* myocardial infarction, *eGFR* estimated glomerular filtration rate, *VHD* valvular heart disease, *LA* left atrium, *RA* right atrium, *IVS* interventricular septal thickness, *LV* left ventricle, *LVEF and RVEF* left and right ventricular ejection fraction, *LVEDVi and RVEDVi* left and right ventricular end-diastolic volume indexed to body surface area (BSA), *LGE* late gadolinium enhancement, *ECV* extracellular volume, *NT-proBNP* N‑terminal pro brain-type natriuretic peptide

^a^among patients with myocardial infarction

### Hematocrit, extracellular volume measurement and statistical analysis

In all patients, venous blood for conventional hematocrit measurement was drawn when placing the i. v. line for contrast agent administration. Standard ECV was calculated using the formula [3]:

$$\text{ECV} = (1-\text{hematocrit}) \times \frac{\left (\frac{1}{\mathrm{T}1\,\text{myo post}}\right)-\left(\frac{1}{\mathrm{T}1\,\text{myo pre}}\right)}{\left(\frac{1}{\mathrm{T}1\,\text{blood post}}\right)-\left(\frac{1}{\mathrm{T}1\,\text{blood pre}}\right)}$$

The population was divided in a derivation cohort, comprising the first 200 consecutive patients. Linear regression analysis was used to create a formula allowing synthetic hematocrit estimation from R1. Synthetic ECV was calculated with the formula stated using synthetic hematocrit instead of conventional hematocrit. The synthetic hematocrit and ECV were then tested in the validation cohort, consisting of the remaining 313 patients. Pearson’s correlation coefficient and Bland-Altman plots were used to describe the association and the agreement between conventional and synthetic ECV. Continuous data are given in mean ± standard deviation and categorical data in percent. Differences between groups were described using the Wilcoxon rank sum test for metric and χ^2^-test for nominal variables. Interobserver variability between native T1-times of LV blood pool measured by two independent physicians (S.A. and B.A.M.) was described by using intraclass correlation coefficients (ICCs). All statistical analyses were performed with SPSS Statistics version 18 (IBM, Armonk, New York) and a level of significance of *p* ≤ 0.05 was prespecified for all tests.

## Results

Among all 513 consecutive patients (mean age 57.4 ± 17.5 years old, 49.1% female) mean conventionally measured hematocrit was 39.9 ± 4.7% (range: 18.3–59.8%), native T1-time of LV blood pool was 1570.6 ± 117.8 ms (range: 1193–1982 ms), and conventional ECV was 28.4 ± 6.8% (range: 18.3–88.2%). The derivation cohort consisted of 200 randomly chosen patients (57.7 ± 17.5 years old, 50.0% female). Baseline characteristics (Table 1) did not differ between derivation and validation cohort. Of note, conventional hematocrit, native T1-times of the myocardium and the blood pool, and conventional ECV were similar among the groups (40.2 ± 4.4% versus 39.9 ± 4.8%, 991.4 ± 54.4 ms versus 990.4 ± 56.0 ms, 1561.4 ± 101.8 ms versus 1576.5 ± 126.7 ms, 28.8 ± 7.8% versus 28.2 ± 6.1%, respectively, *p* > 0.05 for all). Table 1 displays the baseline characteristics stratified by derivation and validation cohort. A significant correlation between R1 and conventional hematocrit (r = 0.592, r^2^ = 0.350, *p* < 0.001) was found in the derivation cohort. By linear regression analysis the following term was derived to best estimate the hematocrit using R1:

$$\text{Synthetic hematocrit}=628.5\times R1-0.002$$

**Fig. 2:**
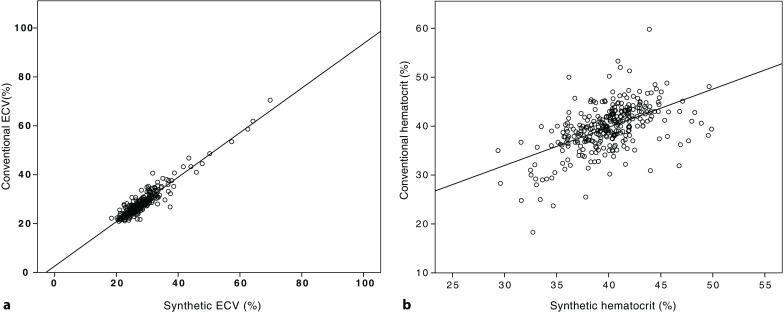
Correlation between synthetic and conventional extracellular volume (ECV) in the validation cohort (**a**) and between synthetic and conventional hematocrits (**b**)

**Fig. 3:**
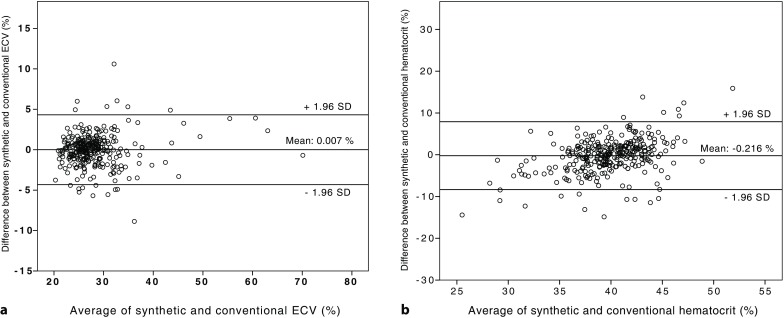
Bland-Altman plot for agreement between synthetic and conventional extracellular volume (ECV) (**a**) as well as between synthetic and conventional hematocrits (**b**) in the validation cohort. Mean difference was 0.007% with limits of agreement between −4.32 and 4.33% for ECV (**a**) and −0.216% with limits of agreement between −8.34 and 7.91% for hematocrit (**b**)

The conventional hematocrit in the validation cohort was similar to the synthetic hematocrit with a close correlation (39.9 ± 4.8% versus 39.9 ± 3.3%, r = 0.533, r^2^ = 0.284, *p* < 0.001). Similar results were obtained with respect to ECV. Synthetic ECV was almost identical to conventional ECV and highly correlated (28.8 ± 6.1% versus 28.0 ± 6.0%, r = 0.943, r^2^ = 0.889, *p* < 0.001). Interobserver variability (two independent investigators) of synthetic hematocrit values and native blood pool T1-time measurements was low with ICCs of 0.899 and 0.849, respectively. In the entire cohort synthetic ECV was 28.3 ± 6.6% and showed an excellent correlation with conventional ECV, which was 28.4 ± 6.8% on average (r = 0.959, r^2^ = 0.920, *p* < 0.001, Fig. 2). By Bland-Altman analysis we found good agreement between conventional and synthetic ECV (mean difference: 0.007%, limits of agreement: −4.32 and 4.33%, Fig. 3).

Treibel et al. [25] proposed a slightly different equation for the calculation of synthetic hematocrit:

$$\text{Synthetic hematocrit}=866.0\times R1-0.1232$$

When using this formula, synthetic hematocrit and ECV in our cohort would be 43.1 ± 4.2% and 27.0 ± 6.8%, respectively, and would result in also significant, yet slightly weaker, correlations with conventional hematocrit and ECV (r = 0.523, r^2^ = 0.274, *p* < 0.001, and r = 0.950, r^2^ = 0.903, *p* < 0.001, respectively).

When stratifying patients according to left ventricular ejection fraction (LVEF < 50% versus ≥ 50%) we found no differences in conventional hematocrit (40.4 ± 5.0% versus 39.8 ± 4.7%, *p* = 0.871), synthetic hematocrit (39.6 ± 3.4% versus 40.1 ± 3.0%, *p* = 0.143), or native blood pool T1-times (1590.2 ± 131.3 ms versus 1568.2 ± 116.0 ms); however, patients with reduced ejection fraction had a higher ECV, both using the conventional (30.7 ± 10.0% versus 28.0 ± 6.2%, *p* = 0.013) and synthetic approach (31.2 ± 9.9% versus 27.8 ± 5.9%, *p* = 0.001).

## Discussion

It has previously been shown that synthetic ECV using blood pool T1-times for hematocrit calculations correlates well with the ECV based on venous blood hematocrit (conventional ECV); however, these data were derived in one single center in the UK. As CMR referral diagnoses, T1 mapping sequences and patient background may differ among countries and centers, we aimed to test the use of synthetic ECV at our institution in 513 patients using a derivation cohort of 200 and a validation cohort of 313 individuals.

The T1-mapping technique has evolved from a research-only tool to a standard sequence that can now be used in everyday clinical practice. It allows a pixel-by-pixel non-invasive quantification of longitudinal (T1) relaxation times. Several T1-mapping methods are currently available [26–28]. For all sequences, satisfactory reproducibility has been reported [29].

So far, several studies have compared results of T1-mapping with histological findings of left ventricular biopsies and found that ECV reflects the amount of diffuse myocardial fibrosis in patients with aortic stenosis [4, 7, 8, 13], hypertrophic cardiomyopathy [4, 10], heart failure [9, 11, 30], dilated cardiomyopathy [6, 12] and mixed patient populations [14]. Furthermore, a small number of studies investigated the impact of T1-mapping on survival and found that higher amounts of diffuse fibrosis are associated with an increased event rate [11, 14, 23, 30–32]. Particularly in infiltrative diseases, T1-mapping has evolved as an important diagnostic tool. In patients with cardiac amyloidosis, native T1-times [16, 33] and ECV [15, 34] were found to often be markedly higher than in other conditions associated with left ventricular hypertrophy [20]. In contrast, due to the low T1-times of fat, low T1-times within the myocardium are a landmark imaging feature in Anderson-Fabry disease where sphingolipids accumulate [20]. Most studies focused on the presence of diffuse fibrosis as the main contributor to myocardial ECV; however, acute or chronic inflammation also alters the extracellular space [35]. It has been shown recently that T1 times are influenced by the presence of myocardial inflammation [36].

One important factor limiting the routine use of ECV in daily practice is the need of the hematocrit for its calculation. Recently, the use of an estimated synthetic hematocrit, based on blood pool T1 time, was proposed [25]. Synthetic ECV showed excellent correlation and agreement with conventional ECV. These results were observed in one single center using one vendor system and it has previously been agreed that T1-mapping results are not directly comparable among centers [37]. Most recently, the same working group reported similar results using different CMR scanners in a pooled analysis of both 1.5 and 3 T [38]. They also investigated synthetic ECV by cardiac computed tomography (CT) and reported excellent results [39]; however, it has to be noted that ECV by CT has so far only been studied to a far lesser extent [40]. Another prospective multicenter study is currently evaluating comparability of T1 mapping results among different CMR units worldwide, using T1-phantoms [41].

In the present cohort we found an excellent correlation between conventional and synthetic ECV by using the formula proposed by Treibel et al. [25]; however, when we used a formula derived from our own patient population, even better agreement between conventional and synthetic ECV was observed. Whether these results are transferable to other centers, which potentially use different T1 mapping sequences and different scanners, remains unknown. Further multicenter trials will be needed to clarify the broad applicability of automatic ECV mapping without the need of conventional hematocrit values.

### Limitations

As a single center study, a certain selection bias must be taken into account when interpreting our data; however, a single center setting ensures consistency in clinical routine, study enrollment, and CMR scanning protocols throughout the study. Differences between the formula by Treibel et al. [25] and ours indicate the need for validation in every single center due to center-specific technical settings. It also has to be mentioned that the correlation between conventional and synthetic ECV in our cohort was weaker compared to the aforementioned study. Of note, factors, such as blood flow, oxygen concentration, and temperature contribute to the relaxation behavior of the LV blood pool [42–48] and may interfere with the results of synthetic hematocrit estimation. Also, the range of hematocrit was narrow in our study (median 40%, IQR 37.1–43.0%), hence extreme values of hematocrit may not be adequately represented. In addition, variability of ECV by repeated measurements has not been performed.

## Conclusion

Synthetic ECV, using native blood pool T1-times to calculate the hematocrit, is feasible and leads to almost identical results in comparison with the conventional method. It may allow fully automatic ECV mapping and thus enable broader use of ECV by CMR T1 mapping in clinical practice.

## References

[1] Pennell DJ (2003). Cardiovascular magnetic resonance: twenty-first century solutions in cardiology. Clin Med.

[2] Wagner A, Mahrholdt H, Holly TA (2003). Contrast-enhanced MRI and routine single photon emission computed tomography (SPECT) perfusion imaging for detection of subendocardial myocardial infarcts: an imaging study. Lancet.

[3] Kellman P, Wilson JR, Xue H, Ugander M, Arai AE (2012). Extracellular volume fraction mapping in the myocardium, part 1: evaluation of an automated method. J Cardiovasc Magn Reson.

[4] Flett AS, Hayward MP, Ashworth MT (2010). Equilibrium contrast cardiovascular magnetic resonance for the measurement of diffuse myocardial fibrosis: preliminary validation in humans. Circulation.

[5] Messroghli DR, Nordmeyer S, Dietrich T (2011). Assessment of diffuse myocardial fibrosis in rats using small-animal Look-Locker inversion recovery T1 mapping. Circ Cardiovasc Imaging.

[6] Aus dem Siepen F, Buss SJ, Messroghli D (2014). T1 mapping in dilated cardiomyopathy with cardiac magnetic resonance: quantification of diffuse myocardial fibrosis and comparison with endomyocardial biopsy. Eur Heart J Cardiovasc Imaging.

[7] Bull S, White SK, Piechnik SK (2013). Human non-contrast T1 values and correlation with histology in diffuse fibrosis. Heart.

[8] Fontana M, White SK, Banypersad SM (2012). Comparison of T1 mapping techniques for ECV quantification. Histological validation and reproducibility of ShMOLLI versus multibreath-hold T1 quantification equilibrium contrast CMR. J Cardiovasc Magn Reson.

[9] Iles L, Pfluger H, Phrommintikul A (2008). Evaluation of diffuse myocardial fibrosis in heart failure with cardiac magnetic resonance contrast-enhanced T1 mapping. J. Am. Coll. Cardiol..

[10] Iles LM, Ellims AH, Llewellyn H (2015). Histological validation of cardiac magnetic resonance analysis of regional and diffuse interstitial myocardial fibrosis. Eur Heart J Cardiovasc Imaging.

[11] Mascherbauer J, Marzluf BA, Tufaro C (2013). Cardiac magnetic resonance postcontrast T1 time is associated with outcome in patients with heart failure and preserved ejection fraction. Circ Cardiovasc Imaging.

[12] Miller CA, Naish JH, Bishop P (2013). Comprehensive validation of cardiovascular magnetic resonance techniques for the assessment of myocardial extracellular volume. Circ Cardiovasc Imaging.

[13] White SK, Sado DM, Fontana M (2013). T1 mapping for myocardial extracellular volume measurement by CMR: bolus only versus primed infusion technique. JACC Cardiovasc Imaging.

[14] Kammerlander AA, Marzluf BA, Zotter-Tufaro C (2016). T1 Mapping by CMR Imaging: From Histological Validation to Clinical Implication. JACC Cardiovasc Imaging.

[15] Banypersad SM, Sado DM, Flett AS (2013). Quantification of myocardial extracellular volume fraction in systemic AL amyloidosis: an equilibrium contrast cardiovascular magnetic resonance study. Circ Cardiovasc Imaging.

[16] Fontana M, Banypersad SM, Treibel TA (2014). Native T1 mapping in transthyretin amyloidosis. JACC Cardiovasc Imaging.

[17] Radunski UK, Lund GK, Stehning C (2014). CMR in patients with severe myocarditis: diagnostic value of quantitative tissue markers including extracellular volume imaging. JACC Cardiovasc Imaging.

[18] Ferreira VM, Piechnik SK, Dall’Armellina E (2014). Native T1-mapping detects the location, extent and patterns of acute myocarditis without the need for gadolinium contrast agents. J Cardiovasc Magn Reson.

[19] Messroghli DR, Niendorf T, Schulz-Menger J, Dietz R, Friedrich MG (2003). T1 mapping in patients with acute myocardial infarction. J Cardiovasc Magn Reson.

[20] Sado DM, White SK, Piechnik SK (2013). Identification and assessment of Anderson-Fabry disease by cardiovascular magnetic resonance noncontrast myocardial T1 mapping. Circ Cardiovasc Imaging.

[21] Puntmann VO, Carr-White G, Jabbour A (2016). T1-Mapping and Outcome in Nonischemic Cardiomyopathy: All-Cause Mortality and Heart Failure. JACC Cardiovasc Imaging.

[22] Puntmann VO, Voigt T, Chen Z (2013). Native T1 mapping in differentiation of normal myocardium from diffuse disease in hypertrophic and dilated cardiomyopathy. JACC Cardiovasc Imaging.

[23] Wong TC, Piehler K, Meier CG (2012). Association between extracellular matrix expansion quantified by cardiovascular magnetic resonance and short-term mortality. Circulation.

[24] Banypersad SM, Fontana M, Maestrini V (2015). T1 mapping and survival in systemic light-chain amyloidosis. Eur. Heart J..

[25] Treibel TA, Fontana M, Maestrini V (2016). Automatic Measurement of the Myocardial Interstitium: Synthetic Extracellular Volume Quantification Without Hematocrit Sampling. JACC Cardiovasc Imaging.

[26] Piechnik SK, Ferreira VM, Dall’Armellina E (2010). Shortened Modified Look-Locker Inversion recovery (ShMOLLI) for clinical myocardial T1-mapping at 1.5 and 3 T within a 9 heartbeat breathhold. J Cardiovasc Magn Reson.

[27] Higgins DM, Ridgway JP, Radjenovic A, Sivananthan UM, Smith MA (2005). T1 measurement using a short acquisition period for quantitative cardiac applications. Med Phys.

[28] Chow K, Flewitt JA, Green JD, Pagano JJ, Friedrich MG, Thompson RB (2014). Saturation recovery single-shot acquisition (SASHA) for myocardial T(1) mapping. Magn Reson Med.

[29] Roujol S, Weingartner S, Foppa M (2014). Accuracy, precision, and reproducibility of four T1 mapping sequences: a head-to-head comparison of MOLLI, ShMOLLI, SASHA, and SAPPHIRE. Radiology.

[30] Duca F, Kammerlander AA, Zotter-Tufaro C (2016). Interstitial Fibrosis, Functional Status, and Outcomes in Heart Failure With Preserved Ejection Fraction: Insights From a Prospective Cardiac Magnetic Resonance Imaging Study. Circ Cardiovasc Imaging.

[31] Ghosn MG, Pickett S, Brunner G (2015). Association of myocardial extracellular volume and clinical outcome: a cardiac magnetic resonance study. J. Am. Coll. Cardiol..

[32] Duca F, Zotter-Tufaro C, Kammerlander AA (2017). Cardiac extracellular matrix is associated with adverse outcome in patients with chronic heart failure. Eur. J. Heart Fail..

[33] Karamitsos TD, Piechnik SK, Banypersad SM (2013). Noncontrast T1 mapping for the diagnosis of cardiac amyloidosis. JACC Cardiovasc Imaging.

[34] Fontana M, Banypersad SM, Treibel TA (2015). Differential Myocyte Responses in Patients with Cardiac Transthyretin Amyloidosis and Light-Chain Amyloidosis: A Cardiac MR Imaging Study. Radiology.

[35] Taylor AJ, Salerno M, Dharmakumar R, Jerosch-Herold M (2016). T1 Mapping: Basic Techniques and Clinical Applications. JACC Cardiovasc Imaging.

[36] Lurz JA, Luecke C, Lang D (2017). CMR-Derived Extracellular Volume Fraction as a Marker for Myocardial Fibrosis: The Importance of Coexisting Myocardial Inflammation. JACC Cardiovasc Imaging.

[37] Moon JC, Messroghli DR, Kellman P (2013). Myocardial T1 mapping and extracellular volume quantification: a Society for Cardiovascular Magnetic Resonance (SCMR) and CMR Working Group of the European Society of Cardiology consensus statement. J Cardiovasc Magn Reson.

[38] Fent GJ, Garg P, Foley JR (2017). Synthetic Myocardial Extracellular Volume Fraction. Jacc Cardiovasc Imaging.

[39] Treibel TA, Fontana M, Steeden JA (2017). Automatic quantification of the myocardial extracellular volume by cardiac computed tomography: Synthetic ECV by CCT. J Cardiovasc Comput Tomogr.

[40] Bandula S, White SK, Flett AS (2013). Measurement of myocardial extracellular volume fraction by using equilibrium contrast-enhanced CT: validation against histologic findings. Radiology.

[41] Captur G, Gatehouse P, Keenan KE (2016). A medical device-grade T1 and ECV phantom for global T1 mapping quality assurance-the T1 Mapping and ECV Standardization in cardiovascular magnetic resonance (T1MES) program. J Cardiovasc Magn Reson.

[42] Piechnik SK, Ferreira VM, Lewandowski AJ (2013). Normal variation of magnetic resonance T1 relaxation times in the human population at 1.5 T using ShMOLLI. J Cardiovasc Magn Reson.

[43] Fullerton GD, Potter JL, Dornbluth NC (1982). NMR relaxation of protons in tissues and other macromolecular water solutions. Magn Reson Imaging.

[44] Lu H, Clingman C, Golay X, van Zijl PC (2004). Determining the longitudinal relaxation time (T1) of blood at 3.0 Tesla. Magn Reson Med.

[45] Shimada K, Nagasaka T, Shidahara M, Machida Y, Tamura H (2012). In vivo measurement of longitudinal relaxation time of human blood by inversion-recovery fast gradient-echo MR imaging at 3T. Magn Reson Med Sci.

[46] Yilmaz A, Bucciolini M, Longo G, Franciolini F, Ciraolo L, Renzi R (1990). Determination of dependence of spin-lattice relaxation rate in serum upon concentration of added iron by magnetic resonance imaging. Clin Phys Physiol Meas.

[47] Wright GA, Hu BS, Macovski A (1991). Estimating oxygen saturation of blood in vivo with MR imaging at 1.5 T. J Magn Reson Imaging.

[48] Silvennoinen MJ, Kettunen MI, Kauppinen RA (2003). Effects of hematocrit and oxygen saturation level on blood spin-lattice relaxation. Magn Reson Med.

